# The Role of Nucleotide Sequencing in Natural Product Drug Discovery

**DOI:** 10.1021/acs.jnatprod.5c00876

**Published:** 2025-09-25

**Authors:** Samantha C. Waterworth

**Affiliations:** Molecular Targets Program, Center for Cancer Research, 70717 National Cancer Institute, Frederick, Maryland 21702, United States

**Keywords:** drug discovery, biodiversity, natural products, biosynthesis

## Abstract

For millennia, humans have drawn upon natural sources for medicinal remedies. However, the discovery process was often a serendipitous endeavor, and the true biosynthetic potential of the planet’s biodiversity remained largely hidden. The advent of nucleotide sequencing offered a pivotal shift, providing tools to decipher the cryptic codes within biosynthetic machinery. In this Perspective, I describe the arc of the sequencing era, from its foundational impact to the current landscape of sophisticated bioinformatic tools. Furthermore, I will address the international legal frameworks being implemented to protect the very biodiversity upon which the field of natural product discovery relies.

## Graphical abstract

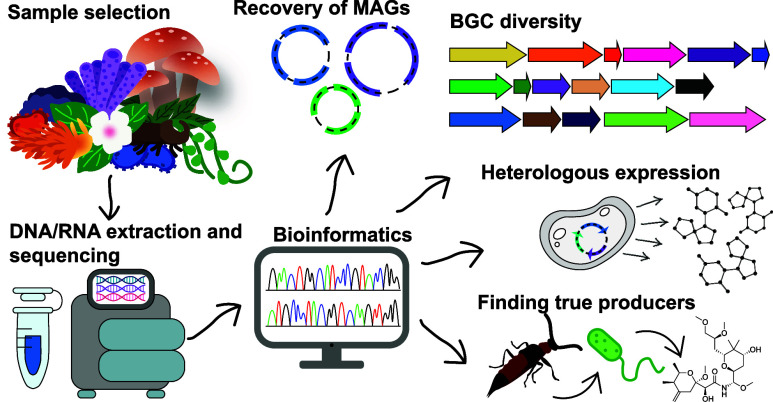

## Introduction

Imagine you knew that incredible treasure lay somewhere over the horizon, and now imagine someone gave you a map to that treasure, and every year, it gets increasingly detailed. You are a creative scientist, how would you use it? Nucleotide sequencing, coupled with advanced computational algorithms, now provides just such a treasure map to the untapped reservoir of natural products hidden in the genetic code of organisms. It offers a direct view into the biosynthetic machinery of life, from microbial players like bacteria and fungi to larger organisms like plants and nematodes.^1–5^ By rapidly identifying BGCs and revealing their evolutionary context and regulatory triggers, genomics has become an indispensable engine for accelerating the discovery, characterization, and understanding of natural product compounds.

My own entry into natural product research, only 12 years ago in 2013, coincided with a field in the midst of a seismic technological shift. My timing gave me a front row seat to the rapid transition away from the methods at the time. My very first projects involved assessing microbial diversity with techniques like denaturing gradient gel electrophoresis (DGGE), a labor-intensive approach of temperamental gels and toxic chemicals. Yet, in what felt like a blink, the entire field pivoted to (relatively) high-throughput amplicon sequencing for assessment of the same bacterial diversity. I vividly recall the steep learning curve: from barely knowing where to find the command-line terminal, I was suddenly navigating it to analyze growing 16S rRNA gene amplicon sequence data sets. This acceleration did not stop. By 2014, I was attempting to assemble my first bacterial genome in search of genes encoding an elusive antimicrobial compound. By 2016, I was evaluating entire metagenomes: hunting for bacteria with a talent for bioactive compound production. The subsequent decade has witnessed an explosion in both sequencing power and sophisticated algorithms that unlock meaning from within that data, expanding the frontiers of natural product discovery. Here, I aim to provide an overview of these tools and technologies, illustrate their applications through case studies, and explore what the future may hold for sequencing in natural product discovery.

## A (Very) Brief History of Sequencing Technologies

The history of sequencing has been reviewed several times,^6–11^ but the pivotal and transformational moment really came in 1977 when Frederick Sanger and colleagues published their method for determining nucleic acid sequences via chain termination with dideoxynucleotides.^12^ A different approach was proposed by Maxam and Gilbert the same year, which was based on the cleavage of DNA,^13^ but owing to its complexity, reliance on hazardous chemicals, and challenges with large-scale implementations, this approach ultimately lost its appeal.^9^ A decade later, the Sanger method was improved upon with the addition of different colored fluorophores for each of the four bases, allowing a new level of automation, efficiency, and speed,^14^ which was initially commercialized by Applied Biosystems.^15^

The second generation, or “next generation”, took advantage of advances in pyrosequencing made by Ronaghi and colleagues,^16,17^ which enabled millions of “short reads” of nucleotides to be sequenced simultaneously. The first commercialization of this approach was made by 454 Life Sciences, later acquired by Roche, in 2006.^18^ For this approach, the nucleic material requiring sequencing needed to be in the form of short, adapter-ligated fragments (generated by PCR amplification or other fragmentation methods) that initially produced reads around 100 nucleotides (nts)^18^—this would improve to around 400 nts as the technology advanced.^19^ IonTorrent^20^ and Illumina^21^ would soon follow with their own technological offerings^10,22^ and would ultimately outcompete the 454 technology, causing its discontinuation in 2013.^22^ Both IonTorrent and Illumina have gone on to extensively improve and expand their platforms and continue to be of significant use today.^10^ Indeed, a brief survey of the NCBI short read archive (SRA) database showed that as of August 19th, 2025, there are 31.2 million Illumina SRA data sets (19.5 million DNA, 11.7 million RNA), and approximately 510,000 IonTorrent SRA data sets (291,104 DNA, 209,171 RNA). Short read data can be either a) “targeted”, where a particular target gene (or gene fragment), such as the bacterial 16S rRNA gene, is amplified and sequenced, or b) “shotgun”, where total DNA or RNA from a given sample is fragmented and sequenced. These approaches will be discussed in greater detail later.

The third generation of sequencing arrived with the advent of “long-read” sequencing, the development of which received the Nature Methods “2022 Method of the Year” award.^23^ Pacific BioScience (PacBio) was the first to bring the sequencing of much larger fragments to the commercial market in 2011, via Single Molecule, Real-Time (SMRT) sequencing.^24,25^ Soon after, in 2014, Oxford Nanopore Technologies (ONT) introduced Nanopore sequencing, which was based on nucleic acids being fed through membrane nanopores and nucleotides being identified via electric current perturbations.^26^ When these long-read technologies initially hit the market, their accuracy could not match that of the short-read options.^27^ To overcome the accuracy limitations of early long-read technologies while leveraging their length, a hybrid approach emerged where accurate short reads were aligned to long-read scaffolds to achieve more accurate sequences.^25,28,29^ More recently reported accuracy is 99.99% with PacBio HiFi sequencing^27,29,30^ and 99.75% with R10.4.1 Nanopore sequencing.^29,31–33^

As sequencing technologies have advanced through different generations, they have not only offered a wider range of capabilities but also generated unprecedented volumes and complexity of data. Navigating this wealth of information, from raw reads to meaningful biological insights, necessitates a sophisticated set of computational algorithms. In the next section, I will explore examples of bioinformatic tools and algorithms essential for assembly, quality assessment, and annotation, among other analyses that allow us to turn a seemingly random collection of A’s, T’s, C’s, and G’s into meaningful results. Please note that throughout this review, I will be focusing on the role of sequencing in the *discovery* of natural product compounds, not the biological screening or reported utility of the resultant compounds.

## Bioinformatic Programs and Algorithms for Biological Insight

### Trimming and Assembly

So, here you are, armed with a verifiable treasure trove of sequence data—so, now what? Whether you sequenced the genome of a single isolated bacterium or the entire genomic cornucopia of an environmental sample, many of the fundamental steps will remain the same (Figure 1). For each step, I will endeavor to provide examples of commonly used or popular bioinformatic tools (Tables 1–3). However, given that the field is constantly evolving, with numerous tools available and new ones continually emerging, it is important to note that this list is not exhaustive, and many other valuable tools exist beyond the ones listed here. Indeed, if the reader is interested in bioinformatics in the context of complex metagenomic shotgun sequence analysis, I would like to direct them to the Critical Assessment of Metagenome Interpretation (CAMI) challenges,^34,35^ in which a wide variety of tools commonly used in metagenome analysis pipelines are compared and discussed. Additionally, while I do outline a possible sequence of tools used for bioinformatic investigation here (Figure 1), the choice and order of bioinformatic analyses should be dictated by the research question(s) at hand and can vary substantially.

**Figure 1:**
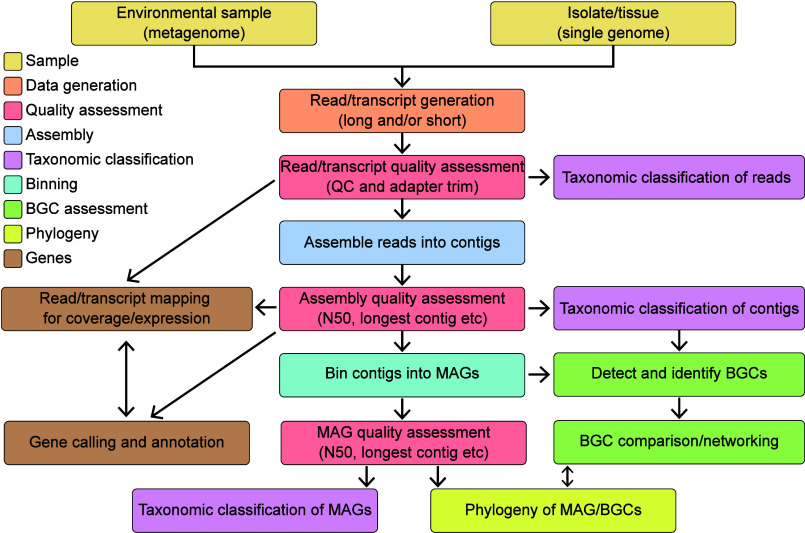
A schematic representation of a possible bioinformatic workflow for using nucleotide sequence data in natural product compound discovery.

**Table 1:** Bioinformatic Tools for Read Preparation and Assembly

Tool name	Read type
Quality assessment and trimming	
Trimmomatic^42^	Short
Cutadapt^43^	Short
FastP^44^	Short
BBDuk^45^	Short
LongQC^46^	Long
NanoPack^47^	Long
LongReadSum^48^	Long
Read assembly	
SPAdes^49^	Hybrid
OPERA-MS^50^	Hybrid
MEGAHIT^51^	Short
NanoPhase^52^	Long
Flye^53^	Long
Canu^54^	Long
Assembly polishing	
Unicycler^55^	Hybrid
Racon^56^	Hybrid
Pilon^57^	Hybrid
PolyPolish^58^	Hybrid
Medaka^59^	Long (Nanopore only)

The first step in sequence analysis is to ensure that you have quality sequence data, and a great example tool for the first step, for short read data, is FastQC.^36^ This algorithm will determine if any sequence adapters are present in your raw sequence data (reads) and the quality of the reads via the Phred score, also known as the quality (*Q*) score. In short, the higher a Phred score, the better the quality of data you have. The accuracy, or error probability (*P*), of a given *Q*-score can be calculated by using the following equation:

$$P=10^{-Q/10}$$

Generally, a *Q*-score of 20 is acceptable as this represents an accuracy level of 99%, but others may opt for a minimum score of 30, which reflects 99.9% accuracy. You can choose between a variety of tools (Table 1) to trim adapters and remove low-quality reads from your data set. The next step is to get the quality reads assembled into contiguous sequences, colloquially referred to as “contigs”. There are a wide variety of assemblers available, some designed specifically for short- or long-read data, while others can generate hybrid assemblies from both data types (Table 1). Additionally, other tools can be used to “polish” assemblies. This can be achieved through mapping of reads onto the assembly (most common) or via kmer-frequency-based approaches.^37^ Independent studies comparing the performance of these tools are available^38–40^ for deciding which tools are best suited to individual research needs. Finally, tools like QUAST^41^ can be used to assess the quality of your assembly.

### Genome Mining for Biosynthetic Gene Clusters

In the context of natural product discovery, after obtaining genomic data from a single isolated bacterium or an entire holobiont metagenome, one could opt to move directly to identifying genes involved in potentially bioactive compound production. This process can be broadly categorized into two approaches: a top-down method, where a known compound has been isolated and the goal is to find the responsible genes; or a bottom-up method, which involves surveying the genes, particularly biosynthetic gene clusters (BGCs), to discover potentially novel chemistry or compounds. One of the most popular tools to achieve this BGC detection is antiSMASH.^60–67^ antiSMASH was first introduced in 2011 ^60^ and is now in its eighth iteration.^67^ The algorithm was initially designed for the detection of bacterial BGCs, but the authors have gone on to include alternative offerings for the detection of fungal and plant BGCs. In addition to antiSMASH, several excellent additional tools for BGC detection exist (Table 2).

**Table 2:** Bioinformatic Tools for Identification and Comparison of BGCs

Tool	Host organism	BGC type(s)
BGC identification		
plantiSMASH^68^	Plants	All
antiSMASH^60–67^	Bacteria	All
DeepBGC^69^	Bacteria	All
PRISM^70,71^	Bacteria	All
EvoMining^72^	Bacteria	All
GECCO^73^	Bacteria	All
BGCProphet^74^	Bacteria and Archaea	All
SMURF^75^	Fungi	All
TOUCAN^76^	Fungi	All
fungiSMASH	Fungi	All
RiPPMiner^77,78^	Bacteria and Fungi	Ribosomally Synthesized and Post-translationally Modified Peptides (RiPPs)
NaPDoS2 ^79,80^	Bacteria and Fungi	Polyketide synthases (PKSs) and nonribosomal peptide synthetases (NRPSs)
BAGEL4 ^81–84^	Bacteria	RiPPs and bacteriocins
RODEO2 ^85,86^	Bacteria	RiPPs
BGC comparison		
BiG-SCAPE^87^	N/A	All
BiG-SLICE^4^	N/A	All
CAGECAT^88^	N/A	All
BGCFlow^89^	N/A	All
SocialGene^90^	N/A	All

Along with these detection tools, robust and reliable databases have been developed to catalogue the growing number of characterized and experimentally validated BGCs, as well as tools to compare newly discovered BGCs with both each other and known reference BGCs. The mainstay of BGC databases has been the Minimum Information about a Biosynthetic Gene cluster (MIBiG) database,^91–94^ with new and expansive databases, such as the Secondary Metabolism Collaboratory (SMC),^95^ BiG-FAM,^96^ The Natural Product Atlas,^97–99^ and BGC Atlas,^100^ as alternative comprehensive and useful resources. Comparative tools for BGCs (Table 2) have been developed to work alongside these databases, enabling the rapid, large-scale comparison of hundreds to thousands of BGCs. This capability allows researchers to efficiently determine the novelty of their BGCs and predict whether they might produce compounds similar to potent, known bioactive molecules. Finally, while not an analysis tool per se, I would be remiss not to mention clinker,^101^ a personal favorite tool for rapid generation of BGC comparison figures, which are publication-worthy straight out of the box, and is now available both online and for local command-line terminal usage.^88^

### Binning and Taxonomic Classification

When BGCs are sourced from a metagenomic sample, one may want to discover which organisms host them and potentially produce valuable compounds. To do this, you can explore binning contigs into metagenome-assembled genomes (MAGs) or taxonomically classifying the specific contigs or MAGs that contain the BGCs you are interested in. The first option is to taxonomically classify the reads or assembled contigs from a metagenomic sample using a variety of tools (Table 3) and helper tools like Taxometer.^102^ The second option is to bin the metagenome into MAGs, followed by their taxonomic classification and further characterization. A wide variety of bioinformatic tools for these processes are currently available (Table 3). Additionally, MAGs can be refined using polishing tools, their quality assessed, and taxonomically classified (Table 3). If there is interest in examining surrounding genes for clues into the ecological role or triggers for BGC expression and compound production, a researcher could use tools for gene calling and annotation (Table 3), and other tools like ARTS^103,104^ or FunARTS^105^ for the identification of self-resistance mechanisms, or perhaps autoMLST,^106^ or PhyloPhlAn^107,108^ for MAG phylogeny.

**Table 3:** Bioinformatic Tools for Binning and Taxonomic Classification

Tool	Input data type	Organism
Taxonomic classifiers		
Kraken2 ^109,110^	Reads	All
RAT^111^	Reads	All
Metabuli^112^	Reads	All
CAT^113^	Contigs	All
MMSeqs2 ^114^	Contigs	All
Tiara^115^	Contigs	Eukaryotes
EukRep^116^	Contigs	Eukaryotes
GTDB-Tk^117,118^	MAGs	Bacteria
CAMITAX^119^	MAGs	Bacteria
MetaShot^120^	MAGs	Bacteria
EUKulele^121^	MAGs	Eukaryotes
Binning tools		
MetaBat2 ^122,123^	Contigs	Bacteria
MaxBin2 ^124,125^	Contigs	Bacteria
Autometa2 ^126,127^	Contigs	Bacteria
MetaBinner^128^	Contigs	Bacteria
CONCOCT^129^	Contigs	Bacteria
AAMB^130,131^	Contigs	Bacteria
SemiBin2 ^132,133^	Contigs	Bacteria
COMEBin^134^	Contigs	Bacteria
McDevol^135^	Contigs	Bacteria
GenomeFace^136^	Contigs	Bacteria
EukFinder^137^	Contigs	Eukaryotes
EukHeist^138^	Contigs	Eukaryotes
MAG polishing tools		
MAGScoT^139^	Contigs and MAGs	Bacteria
BinSPreader^140,141^	Assembly graph, contigs, MAGs	Bacteria
uBin^142^	MAGs	Bacteria and archaea
RefineM^143^	Contigs, MAGs and mapped reads	Bacteria
MAG quality assessment		
CheckM2 ^144,145^	MAGs	Bacteria
EukCC^146^	MAGs	Eukaryotes
BUSCO^147^	MAGs	Eukaryotes
Gene calling and annotation		
Prodigal^148^	MAGs or contigs	Bacteria
Prokka^149^	MAGs	Bacteria
Balrog^150^	MAGs	Bacteria
Bakta^151^	MAGs	Bacteria
MetaEuk^152^	MAGs or contigs	Eukaryotes
AUGUSTUS^153,154^	MAGs or contigs	Eukaryotes

The development of all these tools has been a critical key to bringing sequencing into the world of natural product discovery, where we have now been able to assess the biosynthetic potential of the unculturable (aka mining microbial “dark matter”), activate silent or cryptic gene clusters, and uncover true biosynthetic origins for improved production of compounds.

### RNA-Seq in Natural Product Regulation and Expression

Along with the detection and annotation of the nucleotide sequence of BGCs, it is often advantageous to understand the regulation and expression patterns of BGCs through analysis of transcriptomic data, otherwise known as (bulk) RNA-Seq data. The transcriptomic data can be used on its own or mapped back to a (meta)­genome of interest. The tools and approaches to investigate this will vary extensively depending on the research question(s) to be answered. By and large, the data can be quality controlled and otherwise preprocessed using many of the tools listed for the DNA, but additional, more specialized tools include RNA-SeQC,^155^ RNA-QC-Chain,^156^ and FastqPuri.^157^ If a researcher would like to map their transcripts back to a reference (meta)­genome, options for alignment tools include Bowtie,^158^ BBMap,^159^ and STAR.^160^ Genes can be predicted using tools like Prodigal^148^ (bacterial), FINDER^161^ (eukaryotes), Augustus^153,162,163^ (eukaryotes), and several others.^164,165^ The raw counts of RNA transcripts mapped to these genes can be counted using tools like FeatureCounts^166^ or HTSeq.^167^ If the experimental approach included different conditions, treatments, or time points, one could use tools like DESeq2,^168^ edgeR,^169^ or many others^170–173^ to assess the differential expression of genes across samples. Additionally, new tools like SeMa-Trap are making their way into the field and aim to facilitate the exploration of RNA-Seq data for optimal culture conditions for the production of bioactive molecules from cultured microbes.^174^

## Case Studies of Sequencing in Natural Product Discovery

In this section, I will cover a variety of both seminal and more unusual examples of where nucleotide sequencing has been used to uncover novel BGCs, bioactive compounds, or reveal the true producers of previously isolated natural product compounds. First, we will explore how genomic data from cultured microbes have been used to facilitate the discovery of natural products. Then, we will examine how this same goal is accomplished using more complex metagenomic data to investigate microbes that are not willing or able to grow under laboratory conditions. Finally, we explore a few examples of how transcriptomic data can be used to access novel chemistries.

### Using Genomic Data to Guide Microbial Culture Conditions

An oft-touted axiom is that the vast majority of microbes are recalcitrant to traditional culture methods in the lab.^175–178^ However, the development of sophisticated and imaginative solutions to access the microbial dark matter,^179,180^ such as the iChip,^181,182^ the SlipChip,^183^ using source nutrients,^184,185^ coculture,^186–188^ and other approaches,^189–194^ are gradually knocking that barrier down. An interesting study was performed in 2024,^195^ wherein researchers leveraged genome sequence data from cultivable and uncultivatable MAGs from the order *Burkholderiales* and found, through comparison of genetic features, that genes involved in vitamin B12 biosynthesis and oxidative stress were enriched in the cultivable group. Armed with this, the researchers tested this observation by adding vitamin B12 to their standard culture media and found that new *Burkholderiales* isolates grew.^195^ This genomic-guided isolation should come as exciting news for microbiologists and natural product researchers alike, as comparison of recalcitrant genomes to their readily cultured cousins may ease the way to exploring intractable microbes and the bioactive treasures they may hold.

Although not specifically focused on bioactive compounds, a comparative transcriptomic study on toxin production in cultured *Prorocentrum lima* dinoflagellates demonstrated that phosphate limitation correlated with increased expression of hypothesized toxin BGCs.^196^ This transcriptional finding was confirmed by concurrent toxin content measurements and indicates that comparative transcriptomics can be readily used to optimize the culture conditions to produce bioactive molecules.

### Using Genomics Data for Targeted BGC Silencing to Uncover Minor Metabolites

An intriguing case of difficult bacteria and sequencing coming together is that of clovibactin.^197^ Here, a rare *Eleftheria* bacterium, the same genus that produces teixobactin,^198^ was isolated *in vitro* for the first time following prolonged incubation. Initial inspections of the extract fraction from the *E. terrae ssp. carolina.* yielded known compound, kalimantacin, and a less abundant, unidentified compound. To increase the yield of the less abundant molecule, researchers sequenced the genome of the bacterium via the generation of long-read PacBio sequencing and subsequent assembly with the Hierarchical Genome Assembly Process (HGAP4) assembler,^199^ part of the SMRT Analysis software suite. They identified the kalimantacin-like BGC using antiSMASH^60–67^ in conjunction with the MIBiG database^91–93^ and used this information to silence the BGC through interruption of the first gene in the operon. Silencing of the BGC encoding the abundant, known compound led to the discovery of clovibactin, a distant structural relative of teixobactin that is a more potent antibacterial agent with a different mechanism of action.^197^

### Using BGC Homology to Identify, Target, and Isolate Novel Natural Product Compounds

A striking example of the innovation that is possible with sequencing in natural product discovery is that of WDB002 from *Streptomyces malaysiensis*.^200^ The researchers, Verdine and colleagues, had observed that the two structurally related FDA-approved immunosuppressive molecules, rapamycin and FK506, had “constant” and “variable” regions in their structure (Figure 2), not unlike the constant and variable regions of an antibody.^200^ The constant region of these molecules would bind peptidyl prolyl isomerase FK506-binding protein (FKBP12), while the variable region would determine the target selectivity. Acting together, the FKBP12 and molecule can then allosterically bind the target, stimulating a conformational change which results in inhibition.^201,202^ They reasoned that, given the BGCs encoding these two molecules were similar,^203–206^ they could mine other Actinomycete genomes for related BGCs of modular compounds (i.e., compounds with constant and variable regions). After screening the sequenced genomes of approximately 135,000 actinomycete isolates, the authors found the related “X1” BGC in *Streptomyces malaysiensis* DSM41697. Through cloning of the X1 BGC, a series of analogs were produced that, like rapamycin and FK506, formed a complex with FKBP12. Among the analogs was WBD002, which targeted the human centrosomal protein CEP250 via a coiled-coil domain previously deemed to be “undruggable”.^200^ The WBD002 molecule and synthetic analogs have since been licensed to Gingko Bioworks for finding the properties of WBD002 that enabled the drugging of the “undruggable” coiled-coil and subsequently for development to treat infectious diseases.^207^

**Figure 2:**
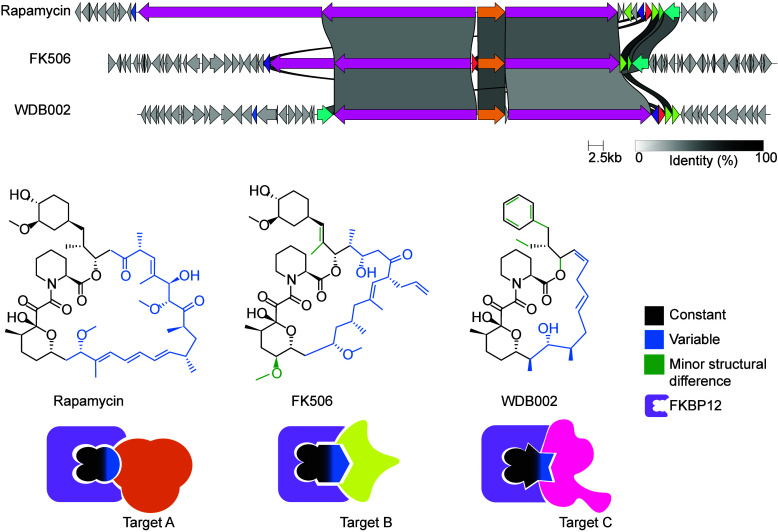
“Modular” compounds with “constant” and “variable” regions are products of related BGCs. Constant regions enable consistent binding to “presenter” proteins (e.g., FKBP12) while “variable” regions dictate target selectivity. The BGC comparison was generated with clinker.^101^ This figure was adapted, with permission from the authors, from Shigdel et al., 2020,^200^ published under a Creative Commons Attribution License 4.0 (CC BY). Copyright 2020 the Author(s). Published by the National Academy of Sciences of the United States of America (PNAS).

### Heterologous Expression of BGCs

Identifying a BGC through sequencing does not always guarantee production in the native host, often due to challenges with standard culture conditions or uncharacterized regulatory pathways. Consequently, researchers may leverage their knowledge of the BGC nucleotide sequence to pursue heterologous expression in a more amenable host organism. Recent examples of this include the discovery of atranorin^208^ and novel bilothiazoles^209^ from complex metagenomes.

Atranorin is a depside produced in a wide variety of lichenized fungi^210^ with an equally diverse array of reported bioactivities.^211–215^ A survey of PKS BGCs detected by antiSMASH in *Cladonia* lichen and related species, clustered using BiG-SCAPE, revealed the presence of a BGC gene cluster family (GCF) exclusively conserved in lichenized fungal species that produce atranorin.^208^ However, at this point in 2021, no one had yet definitively linked a BGC to any known lichen-produced compound. Knowing that prior expression attempts in established fungal heterologous hosts had been unsuccessful, the authors, Kim and colleagues, introduced the putative atranorin BGC genes (*atr*1–*atr*4) into an unconventional host: the plant pathogen *Ascochyta rabiei*, leading to the successful synthesis of atranorin.^208^ The same year, Kealey and colleagues used the sequence data of a BGC from a *Pseudevernia furfuracea* lichen to isolate lecanoric acid, heterologously expressed in an engineered *Saccharomyces cerevisiae*.^216^

The story of the bilothiazoles is particularly intriguing, as the BGC was identified from a metagenomic data set of a human microbiome sample, showcasing the potential for natural product synthesis in our own gut microbiota.^209^ The authors identified thiazol­(in)­e structural motifs from genomes of gut-associated bacteria as a promising lead for bioactivity. Therefore, they opted to focus on NRPS BGCs from two human gut shotgun metagenomic data sets, identified using antiSMASH,^60–67^ that included modules predicted to be involved in heterocyclization. Next, they contrasted their prioritized BGCs into gene cluster families (GCFs) with BiG-SCAPE,^87^ which ultimately led them to focus on the GCF including the novel bilothiazoles (*bil*) BGC. The BGC is predicted to originate from an uncultured *Bilophila* sp. strain, relatives of which are known pathobionts of the human gut, linked to the incidence of disease and cancer.^209^ In this case, the BGC was not cloned from the source organism. Instead, it was produced via *de novo* synthesis, reorganized and codon-optimized, and cloned into an *E. coli* host.^209^ Subsequent induced expression of the BGC resulted in the production of a suite of novel bilothiazoles, which exhibited weak to nonexistent anticancer and antibacterial activity, but did exhibit notable indicators for inhibition of DNA-repair and transpeptidases.^209^

### Using Metagenomics to Reveal the Evolutionary History of Bacterial Symbionts

For many years, the origin of the potent cytotoxic cyclic peptides known as patellamides, isolated from the sea squirt (ascidian) *Lissoclinum patella*, was uncertain. In 2005, the Schmidt group provided a definitive answer via metagenomics, showing that the true producer was the symbiotic cyanobacterium: *Prochloron didemni*.^217^ Using the tblastn algorithm^218^ (using an amino acid sequence query against a nucleotide reference database), the authors concluded that the putative BGC for patellamide comprised seven genes (*patA*–*patG*).^217^ The authors then generated fosmid libraries and used the BGC sequence information to determine which of their clones contained the complete predicted pathway. Heterologous expression of the fosmids resulted in the production of both patellamides A and C, confirming unequivocally that the identified BGC in *P. didemni*. was responsible for patellamide biosynthesis.^217^ An additional suite of compounds, the patellazoles, had similarly been isolated from the *L. patella* tunicate.^219,220^ However, there was no evidence of their biosynthesis in the symbiont *P. didemni* genome. The patellazoles were subsequently hypothesized to be produced by other members of the complex *L. patella* microbiome.^221^ The Schmidt group confirmed this hypothesis by constructing a complete genome of a symbiotic α-proteobacterium: *Candidatus* Endolissoclinum faulkneri from assembled Illumina shotgun metagenomic sequence data.^222^ Within this MAG, a single hybrid NRPS-trans-AT PKS BGC (*ptz*) was detected that matched the predicted biosynthesis of patellazoles.^222^ A remarkable finding was that, unlike the sympatric *P. didemni* symbiont, which included all genes necessary for a free-living lifestyle,^221^ the *Ca*. E. faulkneri symbiont had undergone significant genome reduction. The maintenance of the *ptz* BGC despite the genome streamlining was concluded as indicative of its importance to the symbiotic interaction.^222^ Finally, in the absence of fossil records, the genomic sequence data extracted from the metagenomic assembly were used to estimate the evolutionary divergence of the symbiont and showed that the *Ca*. E. faulkneri symbiont had likely been associated with the *Lissoclinum* tunicate for 6–30 million years.^223^

### Using Metagenomics to Find the True Producers of Pederin-like Compounds

The pederin-like family of compounds has long fascinated me from both biosynthetic and evolutionary perspectives. While largely unified in their potent biological activity,^224^ this family of compounds has been isolated from a remarkably diverse array of organisms, and sequencing efforts have gone on to show that the BGCs responsible for their production stem from an equally wide array of bacterial taxa (Figure 3).

**Figure 3:**
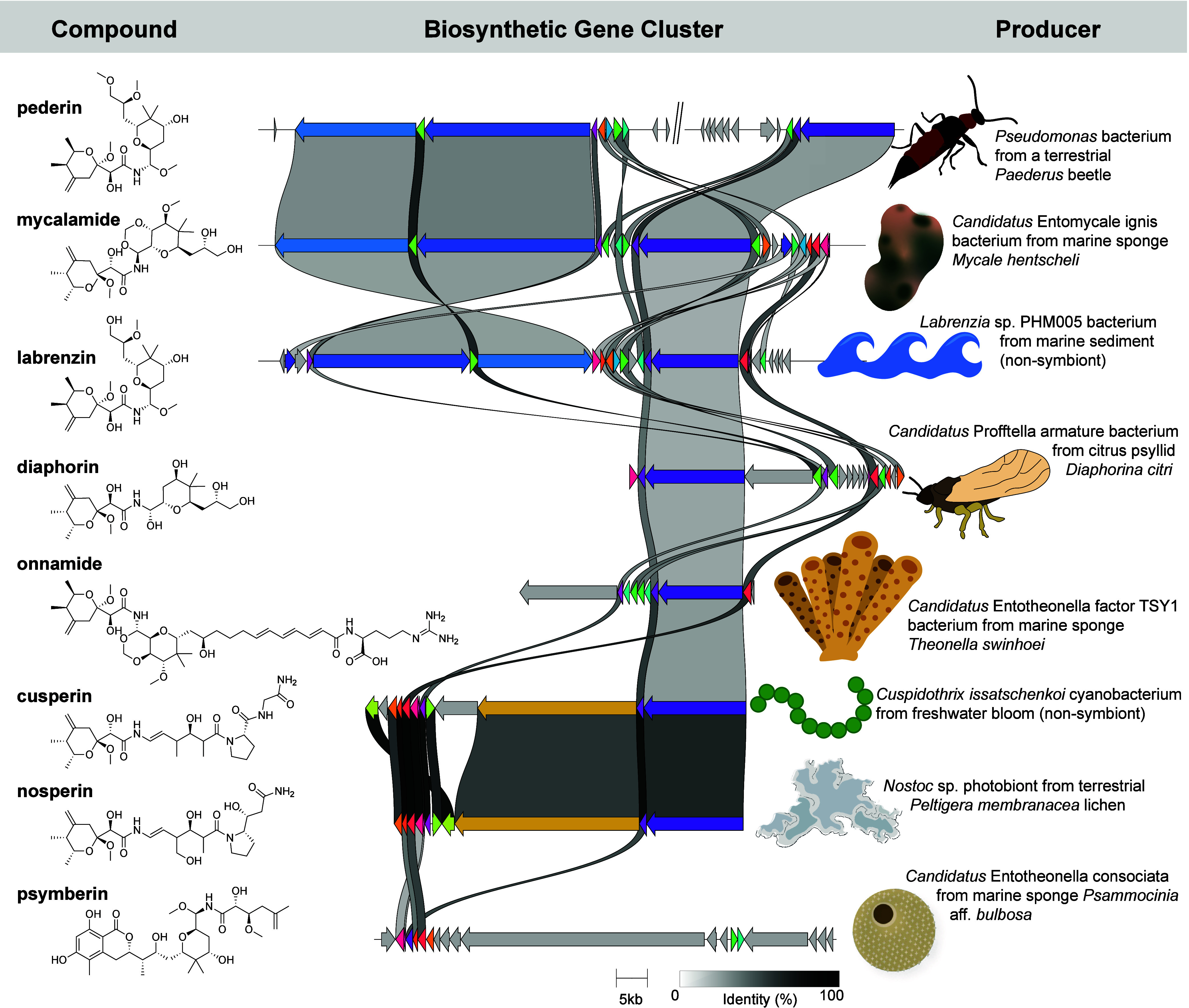
Comparison of the family of pederin-like compounds, their associated BGCs, and the organisms from which they were isolated. Homologous genes are indicated by color, and the level of homology is indicated with a color scale. Comparative BGC visualization was generated using clinker.^101^

Pederin was initially isolated from the *Paederus* fuscipes beetle, and its structure was determined in the early 1900s.^225^ But the BGC (*ped*) would not be described until 2002.^226^ The *ped* cluster was elucidated by the Piel group through the construction of a cosmid library and screening of the library with PCR primers to identify clones with PKS regions.^226^ Sequencing and subsequent assembly of the cloned regions resulted in the recovery of a ∼110kb region including genes *pedA*–*pedH*.^226^ Analysis of genes beyond the *ped* BGC showed that they were most homologous with genes from *P. aeruginosa*, providing the first evidence that the pederin producer was likely a *Pseudomonas* bacterium.^226^ A subsequent study, also using cosmid clones, would reveal additional *ped* genes elsewhere on the symbiont’s genome.^227^

Following this, in 2004, the BGC encoding onnamides, theopederins, and pseudoonnamides was recovered from the yellow chemotype of the marine sponge *Theonella swinhoei* using a similar cosmid library approach.^228^ The producing bacterium was elusive until 2014, when single-cell sorting, genomic amplification, and subsequent sequencing revealed that *Candidatus* Entotheonella factor TSY1 was the true producing organism.^229^ In 2009, the BGC of psymberin (also known as irciniastatin A) was recovered from an unknown bacterium harbored in the marine sponge *Psammocinia* aff. *bulbosa*, through a combination of targeted sequencing of PKS genes using the specificity of the ketosynthase domains as a guide and fosmid libraries.^230^ In 2023, guided by 16S phylogeny analysis, *Candidatus* Entotheonella consociata was proposed as the psymberin-producing bacterium, but a full genome has not yet been sequenced to validate this hypothesis.^231^

In 2013, the BGC of another pederin-like compound, nosperin, was elucidated. This time, the compound would be recovered from a terrestrial *Peltigera membranacea* lichen, and the producing organism found to be its resident *Nostoc* sp. photobiont.^232^ The researchers, Andrésson and colleagues, achieved this by extracting the total genomic DNA from the lichen thallus, sequencing the DNA via both Roche 454 and Illumina sequencing before assembling with an older assembler, MIRA.^233^ PKS genes were searched for using tblastn, and 18 candidate BGCs were recovered, one of which bore striking similarity to the known pederin BGC.^232^ Unfortunately, attempts to recover a pederin-like compound from the lichen tissue were unsuccessful.^232^ However, spurred on by their sequencing evidence, the researchers were not only able to show that the BGC was expressed *in situ* but also were able to culture the *Nostoc* symbiont, confirm the presence of the pederin-like BGC via PCR, and then recover the novel nosperin compound from a 25L culture.^232^

The same year, the BGC for another pederin-like compound, diaphorin, was uncovered. The Asian citrus psyllid, *Diaphorina citri*, was known to harbor two distinct intracellular symbionts: *Carsonella*_DC (a predicted nutritional symbiont), and a second, more enigmatic betaproteobacterial symbiont, which would be later named *Candidatus* Profftella armature.^234^ In an effort to better understand the latter, Fukatsu and colleagues extracted DNA from bacteriomes dissected out from a female *D. citri* specimen.^234^ Sanger sequencing of the shotgun plasmid library and subsequent assembly with an older assembler, Phred-Phrap,^235^ allowed the researchers to recover complete genomes for both intracellular symbionts. Much like the *Ca*. E. faulkneri symbiont from *L. patella* (patellazole producer), the *Ca*. P. armature symbiont genome was drastically reduced but devoted 15% of its 0.46Mbp genome to two disjointed loci of a PKS BGC (*dipA* – *dipT*).^234^ Given the striking similarity between their recovered BGC and that of pederin, Fukatsu and colleagues collected and extracted over 1000 *D. citri* psyllid specimens to isolate and structurally elucidate diaphorin.^234^ Similar to the case of nosperin, without sequence data to tip them off, diaphorin and its potential defensive role in the symbiosis may never have been discovered!

In 2018, Hrouzek and colleagues successfully cultured *Cuspidothrix issatschenkoi*, a cyanobacterium from a freshwater bloom.^236^ An antiSMASH^60,67^ analysis of a draft genome from this bacterium, which was binned from shotgun metagenomic sequencing data from the same bloom, indicated the presence of a nosperin-like BGC.^237^ The researchers subsequently isolated a novel pederin-like compound, cusperin, from the *C. issatschenkoi* culture and confirmed the presence of the cusperin BGC from whole genome sequencing.^237^ Furthermore, by analyzing the cusperin BGC modules, the researchers inferred the absolute configuration of several chiral carbons within the cusperin molecule.^237^ Thus, the sequencing data not only revealed that a new compound was to be found but also proved to be useful in its structural elucidation.

In 2020, Piel and colleagues generated shotgun metagenomic data from the marine sponge *Mycale hentscheli* ^238^ from which mycalamide, a pederin family compound, had been previously isolated.^239^ Illumina sequence data had been assembled with both MEGAHIT^51^ and metaSPAdes,^240^ and the resultant contigs were binned into MAGs with MetaBAT2.^238^ Following quality assessment and taxonomic classification of the MAGs with CheckM^239^ and GTDB-Tk,^239^ respectively, the MAGs were searched for BGCs using antiSMASH.^60,67^ They discovered a BGC predicted to encode mycalamide within a MAG classified within the marine gammaproteobacterium group UBA10353, later designated as *Candidatus* Entomycale ignis.^238^

Finally, a free-living and culturable marine bacterium, *Labrenzia* sp. PHM005, was isolated from marine sediments. Following long-read sequencing and subsequent assembly into a single contig, the genome was assessed with antiSMASH^60,67^ and found to include a pederin-like BGC.^241^ Subsequent genetic manipulation of the BGC allowed the researchers to prove unequivocally that the BGC was responsible for the production of novel pederin family compound, labrenzin, and further provided insights into the functions of genes common to pederin-family BGCs.^241,242^

### Using Transcriptomic Data for Drug Discovery

Transcriptomic analysis of *Aspergillus flavus* fungal strains revealed that global regulatory gene *veA* influenced the transcription of at least half (28 out of 56) of all predicted BGCs in the strains.^243^ The *veA* gene is involved in regulating a variety of processes in fungi, particularly Aspergillus species, that forms the core of the velvet complex, a multiprotein assembly crucial for development and secondary metabolism, primarily in response to light.^244–246^ Using this transcriptional data, Cary and colleagues, found that a novel BGC was expressed most intensely in the fungal sclerotia and subsequent isolation efforts resulted in the identification of a known compound aflavarin.^243^ The researchers additionally showed that the presence of aflavarin increased the production of sclerotia structures, and likely served an additional defensive anti-insectan role.^243^

Another illustrative example of this comes in the form of a study of four cultured *Salinispora* bacterial strains, wherein comparative transcriptomics were used to uncover regulatory mechanisms silencing orphan gene clusters.^247^ An unexpected result of this study was that most BGCs (75%) present in the cultured bacteria were expressed in both exponential and stationary phases of growth—an observation largely at odds with the long-held belief that bioactive compounds are produced largely in the stationary phase and that most BGCs are not expressed under standard culture conditions.^247^ More recently, the finding that secondary metabolites can be produced *en masse* during exponential growth was validated in the myxobacterium *Sorangium* sp. So ce836.^248^ In this work, researchers found, through comparative transcriptomics, that 80% of the detected BGCs were expressed during exponential growth and could be linked to increased bioactive compound production.^248^

The application of transcriptomics for the discovery of plant natural products has helped researchers narrow down which tissues are best targeted for maximum compound recovery. One such example is the study of the tissues of *Panax quinquefolius* (ginseng), which led researchers to the discovery that the greatest concentration of characteristic flavonoids occurred in aerial plant components, such as leaves and flowers.^249^ Conversely, a recent study on the close relative *Panax japonicus* revealed that genes predicted to encode triterpene saponins were most transcribed in root tissues.^250^

Another example is a study of *Ferula assafoetida*, a plant with ties to the mysterious *Ferula* species, the resin of which was the core of some of the earliest medicines for cancer prized by physicians of the ancient world.^251,252^ Here, through contrasting transcriptional data from the various plant tissues, they concluded that the flowers likely had the greatest concentrations of potentially bioactive terpenoids and coumarins.^253^ Subsequently, a second *Ferula* species, *F. feruloides*, was tracked via transcriptomics to determine the optimal time for harvest for the recovery of bioactive compounds, informing future choices for efficient recovery of the compounds of interest.^254^

## Sequencing for Taxonomic Resolution, Dereplication, and Chemotaxonomy

A critical but often overlooked role of sequencing in natural product discovery is the accurate taxonomic classification of organisms and the dereplication advantage associated with it. It is a well-known adage that in the majority of cases, the chances of rediscovery of compounds increase when exploring organisms that are closely related.^99,255–257^ While not without exceptions,^258–260^ there is often a correlation between the relatedness of secondary metabolites and host phylogeny as broad as kingdom level,^261^ as well as within lower taxonomic ranks, with examples in myxobacteria,^262^ Antarctic slugs,^263^ plant-associated fungal endosymbionts,^264^ bacteria,^265,266^ marine sponges^267–270^ and corals,^271^ and various plant species.^272–275^

In addition to reducing the odds of compound rediscovery in closely related organisms, sequence-based phylogeny can also be used directly for bioprospecting natural products.^276^ For example, in one report,^277^ the researchers assessed the polyynes BGC distribution across *Pseudomonas* species, and found a previously unreported, conserved BGC produced a novel polyyne that they named protegencin. The same compound was independently recovered from a different *Pseudomonas* by Murata and colleagues the same year,^278^ and it was later found that this compound could act as an algicide.^279^

In a different study, researchers combined sequence-based phylogeny data with bioactivity for over 16,500 plant-derived compounds from more than 7,500 different plant species from the island of Java to ultimately propose “hot” and “cold” clades that would be more likely to yield antibiotic compounds or compounds with novel structures.^280^ The “hot” clades include native Javan plants within genera *Blumea*, *Carpesium*, *Pluchea*, *Pterocaulon*, and *Sphaeranthus*, where over half the included species produced compounds with antiinfective activities.^280^ In a similar phylogenetic investigation,^281^ the *rbc*L plastid marker was amplified from 2,621 plant species from South Africa, New Zealand, and Nepal, and used to construct phylogenetic trees. The researchers then appended additional metadata about which specimens were used in traditional medicine practices and found that plants utilized in traditional medicine systems, from these three distinct countries, were found to converge in specific phylogenetic “hot nodes”. The authors suggest that because related plants often share similar chemistry, a “hot node” (a lineage with significant medicinal use in one country) can predict the medicinal applications of related species elsewhere. Consequently, they argue that these genera are prime candidates for bioprospecting.

## Natural Product Discovery through BGC Phylogeny

While not dissimilar to pure taxonomic phylogeny-based approaches, assessment of BGC phylogeny has yielded some exciting compounds from a variety of sources. The approach centers around finding clusters of related BGCs, exploring their evolutionary history or relatedness, and using this information to guide or otherwise facilitate production of the encoded molecules (Figure 4). Some of the most popular tools for exploring BGC similarity are BiG-SCAPE^87^ and its sister algorithm for massive data sets, BiG-SLiCE.^4^

**Figure 4:**
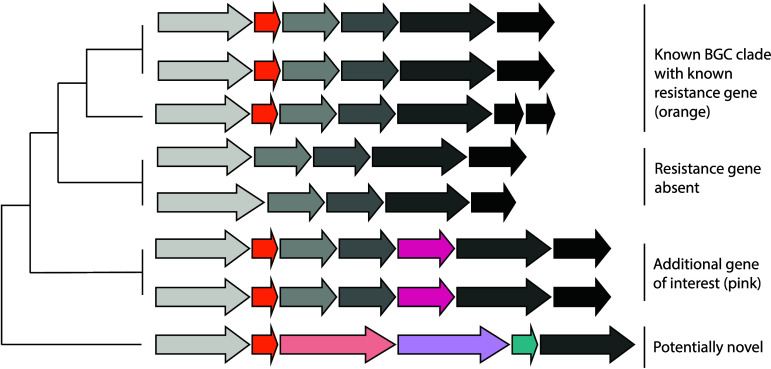
A simplified schematic of the conceptual frameworks guiding the use of biosynthetic gene cluster (BGC) phylogeny for novel compound discovery in the selected case studies.

In 2020, researchers used this approach to isolate a novel glycopeptide antibiotic, named corbomycin, from a *Streptomyces* strain. In this study, the researchers reasoned that by searching for BGCs with no known resistance genes they could uncover molecules with novel mechanisms of action. They built a phylogenetic tree of glycopeptide antibiotic BGCs they had previously identified,^282^ discovered a clade of BGCs that had no known self-resistance genes but did include a BGC for the known compound, complestatin. Corbomycin and complestatin were isolated from *Streptomyces* strains carrying BGCs from this clade and were subsequently found to have a shared, novel mechanism of action, whereby they inhibit autolysins, essential peptidoglycan hydrolases, and subsequently inhibit peptidoglycan biosynthesis.

More recently, the BGC-phylogeny guided approach has similarly resulted in the isolation of the novel polyene macrolide antifungal compound, mandimycin.^283^ The authors of this work undertook the gargantuan task of recovering almost 2 million BGCs from ∼316,000 sequenced bacterial genomes and then parsed them all in search of putative mycosamine-transferring glycosyltransferases, which narrowed the list down to a little under 300 BGCs of interest. Phylogeny of these glycosyltransferase genes revealed a clade of genes distinct from those from the known polyene macrolide antibiotic BGCs. A BGC originating from *Streptomyces netropsis* DSM 40259 within this clade was selected, ultimately resulting in the isolation of the novel, bioactive mandimycin compound. This case study highlights how sequencing data can facilitate and streamline the discovery of novel molecules while simultaneously mitigating the chances of rediscovery.

Finally, a sizable effort from Robey and colleagues^2^ to recover and cluster 36,399 BGCs from 1,037 fungal genomes did not result in the identification of any novel compounds, but did enable the prediction of metabolite scaffolds based on their clustering with known, curated BGCs from the MIBiG database.^91^ Additionally, they noted which BGCs tended to be found across different taxonomic lineages, which would aid in limiting rediscovery. They also went on to show that, contrasting the fungal BGCs they recovered against a set of 24,024 bacterial BGCs, fungi and bacteria occupy different biosynthetic spaces, a trend that was mirrored when they performed a similar clustering of known fungal and bacterial secondary metabolites.^2^ The authors also noted that when assessing the chemical space in which fungal and bacterial compounds are found, the fungal metabolites tend to cluster more closely to FDA-approved compounds and proposed that fungal metabolites may be “more druglike” than their bacterial counterparts. Together, this illustrates that novel compounds have yet to be discovered in fungi as there is enormous orphaned biosynthetic potential.

## BGCs and Natural Products from Unexpected Sources

While the discovery of novel natural products and their associated BGCs is largely considered within the microbial world and plants, more recent discoveries have shown that BGCs can be found across all kingdoms of life. For example, a study of 208 algal genomes used antiSMASH to uncover 2,476 candidate BGCs.^284^ The authors utilized domain organization homology to compare the recovered BGCs to the reference BGCs. They found that algal BGCs had distinct characteristics, with NRPSs favoring LCL condensation domains (unlike those favored by bacteria, which are typically DCL/Dual domains), and PKSs were structured iteratively, similar to those in fungi.^284^

Even animals prove to be unexpected sources. The nematode *Caenorhabditis elegans* produces nemamides, which are hybrid polyketide-nonribosomal peptides encoded by a BGC within the worm’s own genome, not a symbiont.^5,285^ In 2003, researchers used transcriptomic sequencing data from purple sea urchin (*Strongylocentrotus purpuratus*) embryos to discover that an endogenous polyketide synthase (PKS) BGC is responsible for the production of its characteristic pigment, echinochrome.^286^ More recently, scientists sequenced five octocoral genomes, identifying a BGC that is conserved across diverse taxonomic families and is essential for producing a cembrene-B-γ-lactone, a core structure central to the biosynthesis of briarane diterpenoids.^287^ Perhaps most surprisingly, a study of common budgerigars (budgies) demonstrated that a single amino acid substitution in a polyketide synthase was responsible for the phenotypic switch from yellow to blue feathers. Researchers confirmed this by expressing the PKS in yeast, which resulted in the accumulation of a yellow pigment.^288^

These discoveries illustrate the enormous biosynthetic potential that lies beyond our conventional comfort zones. Through sequencing and subsequent identification and heterologous expression of BGCs from diverse and complex organisms, we can begin to uncover an even wider array of chemical scaffolds than previously imagined.

## Peering into the Future: Technologies and Algorithms to Look Out For

A brand new tool on the block, aimed at high-throughput detection of BGCs, is BGCProphet.^74^ It utilizes a transformer-based language model that treats genes as “words” within a genomic context, allowing it to “learn” the syntax and vocabulary of BGCs from known examples. The developers posit that this method can identify novel BGC types missed by rule-based finders like antiSMASH and demonstrated its scalability by screening over 90,000 genomes and MAGs to identify hundreds of thousands of putative BGCs. This approach is undoubtedly promising for uncovering novel BGCs, but some aspects of the initial report warrant careful consideration. A key concern relates to the model’s training and benchmarking. Using antiSMASH to define the negative training set (genomes with antiSMASH-defined BGCs removed) may have inadvertently contained novel BGCs that antiSMASH cannot detect, potentially biasing the model against identifying the very targets that it aims to find. Furthermore, although BGCProphet identifies a greater number of BGCs than established tools, the report lacks the experimental validation needed to confirm that these additional predictions are genuine. This, personally, raises questions about the model’s specificity and its ability to distinguish true BGCs from other colocated gene clusters with similar organizational features, such as operons involved in primary metabolism. Ultimately, BGCProphet represents an intriguing and powerful application of AI for BGC discovery, but the true test of its utility will be the experimental validation of its predictions by the natural products community. For now, BGCProphet could be viewed as a promising complementary tool whose novel predictions, when used in tandem with established methods, could reveal previously hidden biosynthetic potential.

One of the most compelling technologies currently being developed is spatially resolved metagenomics and metatranscriptomics. As the biosynthesis of bioactive natural products often occurs in very specific niches or microenvironments (e.g., soil aggregates, particular host tissues, and biofilms), spatial investigation could provide critical insights into the ecological underpinnings that trigger the production of these compounds. This could lead to significant advancement in cultivation strategies or activation approaches *in vitro*. In a recent elegant series of experiments,^289^ a team of researchers investigated how a plant host (*Arabidopsis thaliana* leaves) and its harbored microorganisms interact through the sequencing of short-read data captured on arrays with a variety of probes (i.e., a mix of probes for 16S, 18S, and ITS rRNA sequences, and poly d­(T) probes for untargeted host mRNA sequences). At a spatial resolution of 55 μm, the researchers could survey the *A. thaliana* host response to the density and diversity of microbial “hotspots” on leaf samples.^289^ For example, when the researchers artificially infected a leaf with a *Pseudomonas* pathogen, they observed a colocalized increase in plant host immune response to the localization of the pathogen. Further, they used this approach to map the distribution of bacterial and fungal taxa on leaves of outdoor-grown *A. thaliana* plants and found that the native bacteria and fungi occupied distinct sections of the leaves (with some overlapping areas) and that the plant host expressed a higher level of defense-related genes in these regions. Although this study did not explore BGCs, the approach paves the way for investigating their spatial expression. Incorporating probes for conserved regions of PKS/NRPS genes, for example, could reveal how BGCs are expressed across host tissues and whether host factors, microbial density, or microbial activity influence their activation.

Finally, an exciting new platform for natural product discovery, published by researchers from the University of Illinois Urbana–Champaign in March 2025, is FAST-NPS.^290^ The FAST-NPS pipeline covers all stages, from genome mining and BGC prediction to heterologous expression and product isolation. The key innovation of the pipeline is utilizing self-resistance genes for BGC prioritization (as used in previous studies, one of which was described earlier in this review) and as a proof-of-concept they used their pipeline to screen 11 *Streptomyces* species, clone 105 BGCs into heterologous *Streptomyces lividans* hosts, leading to the recovery of 23 compounds of which eight had either antibacterial or antineoplastic activity.^290^ The pipeline utilizes two existing tools: antiSMASH^60–67^ for BGC detection, and ARTS^103–105,291^ to detect self-resistance genes. However, the platform’s general applicability is limited by its use of the iBioFAB^292^ robotics system, which automates the CAPTURE process^293^ for capturing, cloning, and heterologous expression of BGCs, and this aspect of the platform may not be readily accessible to all researchers. Nonetheless, researchers with limited resources may still be able to apply many of the strategic approaches employed by the FAST-NPS developers to create an effective, albeit lower-throughput, version of the platform.

## Emerging Regulatory Framework for Access to Digital Sequence Information

In an effort to protect the rich biodiversity often stewarded by developing countries, the Convention on Biological Diversity (CBD) was amended in 2014. The Nagoya Protocol on Access to Genetic Resources and the Fair and Equitable Sharing of Benefits Arising from their Utilization (generally referred to as the Nagoya Protocol) established guidelines for access to these materials and resources.^294,295^ Simply put, if researchers or companies aim to use a genetic resource, they need to obtain permission from the country of origin and/or community from which that resource originated as well as agree to share any benefits gained from the resource with the originating country or community. At the time of writing this review, 142 parties have ratified the protocol (i.e., formally agreed to be bound by the protocol). While the protocol was initially drafted with physical, biological samples in mind, there have been subsequent efforts to now include digital sequence information (DSI), or genomic sequence data (GSD) within the scope of the protocol.^296,297^ While these two terms are not entirely synonymous, there is significant overlap between these two concepts, and I will use the acronym DSI with the intent of discussing the topic with both DSI and GSD in mind.

Given that a significant percentage of approved drugs are either natural products or a derivative thereof,^298^ and that natural products have been reported to lead to an increased likelihood of clinical success,^299^ the Nagoya protocol should be considered in the context of ethical natural product drug discovery. Additionally, given the increasing role that sequencing data may play in the future of natural product drug discovery, the topic of the use of DSI requires a nuanced consideration. The subject has been comprehensively reviewed by several experts,^296,297,300–303^ but I will briefly summarize the key points here.

While open access to DSI for research purposes is lauded as a positive boon for the scientific community, aiding global efforts to rapidly address challenges and facilitating innovation, the advent and subsequent advancement of both DNA sequencing and synthesis technologies have led to fears of a potential digital version of biopiracy.^296,304–306^ In short, access to nucleotide sequences can negate the need to travel to a country, acquire necessary permits, set up sharing agreements, and physically collect a sample. Instead, nucleotide sequences can be harvested online and synthesized before being cloned into an appropriate host. This can bypass agreement mechanisms and, by extension, the legal requirements for benefit-sharing. Currently, the actions of delegates tasked with finding a solution to this problem are lagging behind the rapid pace at which sequencing, data transfer, hosting, and distribution technologies are evolving.^296,301^ Compounding challenges due to a rapidly shifting technological environment, the exact definition of DSI has not yet been agreed upon.^297,301^ Even if the definition of DSI were defined, strategies to ensure compliance in the digital age are a significant hurdle.^296^

While different solutions have been proposed,^296^ the Conference of the Parties (COP) meeting 15 (COP15) resulted in the proposal for the establishment of a global fund, among other strategies, to address these issues.^301^ The goal was for countries to have negotiated terms to achieve this by October 2024, with guidelines stating that the multilateral mechanism by which benefits are shared must not hinder research or innovation, allow open access data sharing, but at the same time provide reliable legal provisions, and generate more benefit than cost.^297,307^ In response, following COP16, the Cali Fund for the Fair and Equitable Sharing of Benefits from the Use of Digital Sequence Information (the “Cali fund”) was launched in February 2025.^308^ Significant hurdles and details remain to be worked out,^258^ but natural product researchers should be aware of these and other^262^ ongoing initiatives, their potential effects on research, and contribute their expertise where possible. Moving forward, continued international cooperation and innovative mechanisms will be crucial to both encourage research and discovery and enable equitable benefit-sharing.^309^ A future without robust conservation is a future with dwindling biodiversity and, in turn, a significantly impoverished landscape for natural product discovery.

## Conclusion

From the foundational Sanger method to revolutionary next-gen technologies, sequencing has steadily revealed the hidden chemical potential encoded within diverse organisms. Marching in lockstep with the ever-advancing technologies are the algorithms for quality control, contig assembly, and crucially, for the targeted mining of biosynthetic gene clusters (BGCs), which have become important technologies for a natural product researcher. These tools not only enable the efficient identification of BGCs but also facilitate large-scale comparisons, allowing researchers to quickly assess the novelty and potential bioactivity of newly discovered gene clusters. In this way, sequencing has been instrumental in unlocking the “microbial dark matter”, revealing the vast biosynthetic capabilities of previously unknown and unculturable organisms. Beyond the identification of BGCs, transcriptomic analyses have provided a critical window into the expression and regulation of BGCs and their associated natural products. This allows researchers to optimize culture conditions for maximal compound production, prioritize promising microbial strains, and even challenge long-held assumptions about the timing of secondary metabolite production. As sequencing technologies continue to advance in accuracy, speed, and affordability and as bioinformatic tools become even more sophisticated, the future of natural product compound discovery promises an exciting era of exploration. The integration of “omics” data, artificial intelligence, and synthetic biology, all underpinned by the power of nucleotide sequencing, will undoubtedly lead to a cascade of discoveries that will invigorate and advance the field of natural product discovery for years to come.
